# IL-2 Expression and T lymphocyte Phenotyping in Young Children Suffering from Upper Respiratory Tract Infection with *Streptococcus Pyogenes*

**Published:** 2016-06

**Authors:** Eda Guadalupe Ramirez-Valles, Verónica Dayali Gutierrez-Martinez, Maribel Cervantes-Flores, Estela Ruiz-Baca, Cosme Alvarado-Esquivel

**Affiliations:** 1 Faculty of Chemical Sciences, Juárez University of Durango State. Avenida Universidad S/N. 34000 Durango, Dgo, Mexico;; 2 Faculty of Medicine and Nutrition, Juárez University of Durango State. Avenida Universidad S/N. 34000 Durango, Dgo, Mexico

**Keywords:** IL-2 expression, phenotyping, T lymphocytes, Streptococcus pyogenes, infection

## Abstract

T cells are components of adaptive immunity and are involved in the resolution of respiratory infections, which are a major cause of morbidity and mortality in young children worldwide. Activation and differentiation of T cells is given mostly by the cytokine IL-2. This study aimed to determine the phenotype of T cells and IL-2 expression in children suffering from upper respiratory tract infection with *Streptococcus pyogenes (S. pyogenes*). For this purpose, IL-2 expression at its gene and protein levels and quantitation of CD4^+^ and CD8^+^ T lymphocytes were assessed in children aged 0-5 years old suffering from upper respiratory tract infection with *S. pyogenes* and healthy children of the same age. Children with *S. pyogenes* infection had a higher expression of IL-2 gene and a lower level of this cytokine expression at protein level than healthy children. The numbers of CD4^+^ T lymphocytes were similar among the groups. In contrast, difference in the numbers of CD8^+^ T lymphocytes among the groups was found. We conclude that infections by *S.* *pyogenes* in young children lead to an increased expression of IL-2 mRNA.

## INTRODUCTION

T cells belong to the adaptive immune system and perform a wide range of functions in immune regulation, inflammation and protective immune response (1). The maturation of these cells is subjected to positive and negative selection to produce CD4^+^ and CD8^+^ lymphocytes. At maturation, lymphocytes leave the thymus, in that moment are considered naive cells until they are activated by signals, then they start to proliferate and differentiate into effector cells (helper and cytotoxic). The activation of naive T cells in the peripheral immune system is the first step of the adaptive immune response (2).

The balance between the differentiations of T cells may be influenced by the types of dendritic cells that initially respond to infections, the cytokines they secrete when are activated by the microorganisms and thus, the type of effector T cell induced (3).

Interleukin 2 (IL-2) is a cytokine secreted by T cells, it regulates the proliferation, differentiation and survival for the cells that produce it (4) also stimulates growth and differentiation of B lymphocytes, NK cells (“natural killer”), LAK cells (“lymphokine-activated killers”), monocytes, macrophages and oligodendrocytes (5).

*Streptococcus pyogenes* (*S. pyogenes*) is a pathogen responsible for at least 616 million cases of respiratory tract infections per year worldwide and 111 million infections in the skin (6), causes 700,000 cases of invasive infections (7) and, in total, cause 163,000 deaths per year (8). The bacteria are phagocytized and destroyed by the macrophages and dendritic cells, is processed into small fragments, attached to MHC II and presented to helper T lymphocytes (ThL) (9).

In this study, the expression of IL-2 at its gene and protein levels and the phenotyping of CD4^+^ and CD8^+^ T lymphocytes were investigated in children aged 0-5 years old with *S. pyogenes* infection and healthy children of the same age.

## MATERIALS AND METHODS

### Selection and description of participants

Through a case-control study, the expression of IL-2 and phenotyping of CD4^+^ and CD8^+^ T lymphocytes were examined in children with *S. pyogenes* infection (cases) and children without *S. pyogenes* infection (controls). The children studied attended the Hospital “Santiago Ramón y Cajal” of the Institute for Security and Social Services of the State Workers and the Health Center No. 1 “Dr. Carlos León de la Peña” of the Secretary of Health in Durango, City, Mexico from June 2012 to September 2013. Inclusion criteria for cases were children with *S. pyogenes* infection, aged 0-5 years old, of any gender. Inclusion criteria for controls were healthy children without *S. pyogenes* infection, aged 0-5 years old (age-matched with cases), of any gender. During the study period, 604 children aged 0-5 years old were tested for *S. pyogenes* infection in the participating Hospital and Health Center. A throat swap sample was obtained from each participant and cultured on a sheep blood agar plate. Identification of *S. pyogenes* was performed by using the VITEK 2 automatic system (bioMérieux, Marcy l’Etoile, France). Of these 604 children, 31 were positive for *S. pyogenes* infection.

Children with *S. pyogenes* infection were aged 0.5-5 years old and included 16 male and 15 female. The control group included 31 clinically healthy children without *S. pyogenes* infection matched for age and sex with cases. Peripheral blood samples from cases and controls were collected by venipuncture using EDTA vacuum tubes (Vacutainer). Whole blood and plasma aliquots were obtained and stored at 4°C and -20°C, respectively until analyzed.

### Expression of IL-2 at gene level

This expression was assessed by RT-qPCR. To achieve this, total RNA was extracted using protocols by Chomczynski and Sacchi (10) TRIzol extraction (Ambion). The RT-qPCR was performed from extracted RNA using the RT-PCR System^TM^ (Promega) commercial kit under supplier conditions. Gene expression of IL-2 was compared to the expression of a constitutive gene (GAPDH) showing relative expression. The oligonucleotides used in this study are shown in Table 1, oligonucleotides for the amplification of IL-2 were designed using the primer quest tool from IDT page using the identification number of the gene bank K02056.1; oligonucleotides for amplification of GAPDH were designed according to sequences reported by Chen *et al*. (11).

**Table 1:** Sequences of the oligonucleotides used for RT-qPCR

Target mRNA	Sequence	Amplicon length (bp)

IL-2	5’ TCC CAA ACT CCA TCA CCT TTC 3’ 5’ CAC CTG AGT CCC TTG CAT ATT 3’	355
GAPDH	5’ TGA ACG GGA AGC TCA CTG G 3’ 5’ TCC ACC ACC CTG TTG CTG TA 3’	306

### Quantification of IL-2 at protein level

To quantify IL-2, the commercial ELISA kit PeproTech human IL-2 was used under supplier conditions. Readings at 5 minute intervals were performed from 0 to 45 minutes after adding the enzyme substrate. Readings were taken at 405 nm and 650 nm.

### Phenotyping of T CD4^+^ and CD8^+^ lymphocytes

These two populations of cells were quantified from whole blood samples by flow cytometry, using reagents, controls and BD FACSCount^TM^ software for whole blood, searching the surface antigens CD3^+^/CD4^+^ and CD3^+^/CD8^+^, according to the supplier conditions.

### Ethical aspects

This study was approved by the Ethics Committee of the Hospital “Santiago Ramón y Cajal” of the Institute for Security and Social Services of the State Workers in Durango City. Information about the study procedures was given to the parents of the children. An informed consent was obtained from all parents of the participants.

### Statistical analysis

The normality of data was assessed by the Kolmogorov-Smirnov test. F test was used for analysis of variance and finally, the data were analyzed using the student’s *t* test. Statistically significance was set at a *p*<0.05.

## RESULTS

### IL-2 expression

**Figure 1:**
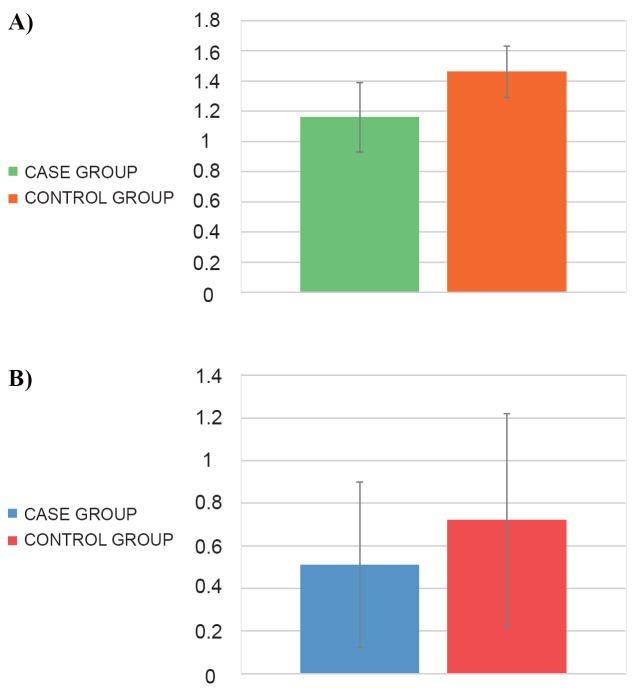
IL-2 expression in the study groups. A) Gene expression; B) Protein expression (ng/ml).

Quantitative analysis by RT-qPCR revealed that the expression of IL-2 was lower (1.16 ± 0.23) in *S.* *pyogenes *infected individuals than in controls (1.46 ± 0.17) (*p*<0.001) (Figure 1A). The protein expression of IL-2, analyzed by ELISA, shown an increased expression in uninfected individuals compared to infected individuals (0.72 ± 0.5 and 0.51 ± 0.39 ng/ml, respectively; *p*=0.032) (Figure 1B).

### CD4^+^ and CD8^+^ T lymphocyte phenotyping

**Figure 2:**
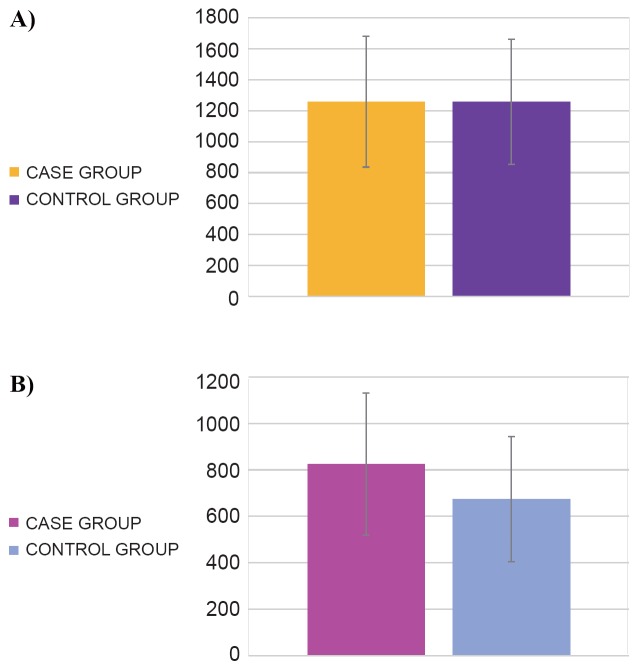
Results of T lymphocyte quantitation in the study groups. A) CD4^+^ T cells (cells/µl); B) CD8^+^ T cells (cells/µl).

No difference in the numbers of CD4^+^ T cells in subjects with *S. pyogenes* infection and controls (1258.3 ± 422.9 and 1257.7 ± 403.4 cells/µl, respectively; *p*=0.48) was found (Figure 2A). In contrast, cases had a higher number of CD8^+^ T cells than controls (824.5 ± 306.3 and 674.1 ± 269.6 cells/µl, respectively; *p*=0.022) (Figure 2B).

## DISCUSSION

In this study, according to the analysis results, there is evidence of a statistically significant difference in the rate of IL-2 gene expression, protein expression, and number of CD8^+^ T cells between infected children with *S. pyogenes* and control individuals. However, under the same statistical conditions, there is no significant difference in the quantitation of CD4^+^ T cells. A study carried out with monocytes from healthy donors which were stimulated with a strain of *S. pyogenes* isolated from children with bacteremia revealed that infection by this bacteria induces the expression of IL-2 messenger RNA (mRNA) and other cytokines (12). According to this finding, the present study shows an increased expression of this cytokine mRNA in samples of patients with this pathogen infection in comparison with those which were free of it. It is expected that because of this infection and with the results showing the expression of IL-2 mRNA had a higher product of protein expression in plasma, however, although a statistically significant difference was found, the highest expression of this cytokine in plasma was present in the infection-free samples. Gene expression can be controlled at various stages, which are divided into: “control at transcription level”, “control at processing level” and “control at translational level” (13). The control of gene activity has as general purpose: the organism can adapt to the properties of various cell types for their benefit (14). A key point in the regulation of gene expression is the control in the cytoplasm of the mRNA translation. In the last decade, micro-RNA (miRNA) and siRNA (small interfering RNA) have emerged as important regulators of translation and elimination of mRNAs (15), more than 800 individual miRNAs have been identified in humans, it is estimated that they regulate 74-92% of mRNAs (16) and these repress translation by several mechanisms including: inhibition of translation initiation, inhibition of elongation in the translation, premature translational termination and co-translational degradation of proteins, there is evidence of an expression of these miRNAs in bacterial infections (17). This may explain somehow the fact that in samples with *S. pyogenes* infection there is a smaller amount of protein in plasma in relation to the samples from healthy individuals, however, further studies are required to endorse these possible explanations for these results.

It has been documented that in infection with *S. pyogenes*, specifically by its toxin A, the mayor histocompatibility complex type II (MHC II) is expressed on cell lines involved in immunity (18). This molecule is expressed on B lymphocytes, dendritic cells and monocytes/macrophages and is responsible for presenting antigens to CD4^+^ T helper cells (Th cells) (19). Therefore, it was expected to find a greater number of these lymphocytes in samples from infected individuals. Besides, *S. pyogenes* is an extracellular bacterium (20, 21) and its protein antigens activate Th cells (3). However, in the present study, there was no difference in the numbers of CD4^+^ T cells between cases and controls.

Although *S. pyogenes* is considered as extracellular, it has mechanisms that allow it to be found within epithelial, endothelial and within neutrophil cells, this is due to its pathogenicity factors (fibronectin binding proteins), but these internalization factors have not been completely elucidated (22, 23), which explains only in part, the fact of a greater amount of cytotoxic CD8^+^ T cells (CTL). It was reported that when the microorganisms are in the interior of cell, the infection should be eradicated through CTL elimination of infected cells (3). Furthermore, in previous studies of children with pneumonia, researchers found bacterial-viral co-infections, especially respiratory syncytial virus, rhinovirus, human bocavirus, metapneumovirus, parainfluenza and influenza viruses co-existing with bacteria such as: *Streptococcus pneumoniae*, *Haemophilus influenzae, Moraxella catarrhalis,* and *S. pyogenes* (24). The samples of the present study were further analyzed to detect a co-infection with both respiratory syncytial virus and the human adenovirus (25), however, this coinfection was not found. A possible co-infection with influenza virus cannot be excluded. In a previous study, it was found that mortality by *S. pyogenes* was associated with an additional infection with influenza virus (7). Besides, in studies of mice, researchers also found this bacterial-viral co-infection (26). It has been reported that transmission of influenza virus requires certain characteristics of humidity and temperature and its incidence is related with wintertime and in rainy season (27). It is important to note that sample collection mostly occurred in the July to September period, corresponding to the rainy season. Therefore, it cannot be discarded the possible association of *S. pyogenes* with the influenza virus, due to the season; thus it could also explain the high amount of CTL in *S. pyogenes* infection. Two assumptions may explain the lower amounts of Th cells found: that *S. pyogenes* resides intracellularly, or a viral co-infection. In both cases, the response would be mostly by CD8^+^ T cells.

## CONCLUSIONS

We conclude that infections by *S. pyogenes* in young children lead to an increased expression of IL-2 mRNA. Contrary to expectations, a lower protein expression of this cytokine was found in samples of children infected with *S. pyogenes*. In addition, there was a greater amount of CD8^+^ T cells and a minor amount of CD4^+^ T cells. Certainly more studies to identify a possible control of mRNA translation and to reveal whether different viruses may be associated with infection by *S. pyogenes* are needed.
